# Abstract of the Proceedings of the Buffalo Medical Association

**Published:** 1862-08

**Authors:** J. F. Miner

**Affiliations:** Secretary


ART. II.—*Abstract of the Proceedings of the Buffalo Medical Association.*

TUESDAY EVENING, JULY 8, 1862.

Present—Prof. James P. White, President, in the Chair, Drs. Samo, Eastman, Congar, Rochester, King, Miner, and Morrell of Niagara.

*Dr. Rochester* reported, that he had met with a case of presentation of the cord, which he had failed to reduce by Thomas' postural method. On the 4th of July, a very stout woman was taken with her seventh labor.—When the patient was first seen by Dr. Rochester, the uterus was well dilated and a large bag of waters protruded. The membranes ruptured spontaneously and an enormous discharge of the liquor amnii brought down three folds of the funis, arranged in parallel lines. The head, vertex presentation, completely filled the dilated os. The patient was immediately placed upon her knees and *breasts.* and repeated efforts were made to carry up the cord, but in vain, it was impossible to push up the head or to get the cord above it. The woman was very large, the room very small, and the day very hot, and she could not keep the prone position but a few moments at a time. The uterine pains were very slight, it would take at least half an hour to obtain a pair of forceps, and ergot was at hand. As far as the mother was concerned, everything favored its administration, and Dr. Rochester thought it would also afford a good chance of viability to the child. In twenty minutes ergotic action was established, in five minutes more the child was born. It was resuscitated by dashes of hot and cold water, and by Marshall Hall's method, and is now strong and healthy.

*Dr. Rochester* said, that the treatment was not such as would generally be advised, but he thought it the best he could adopt under the circumstances, and the event was all that could be desired.

*Dr. Eastman* remarked, that in the case reported by him a few months since, it would be recollected that position alone was not sufficient to restore the cord: the hand was carried up and the cord replaced in the uterine cavity, past the head of the child, when pain immediately succeeding, caused the head to descend and occupy the os-uteri. Dr. E., commented upon the practice adopted by Dr. Rochester, and regarded it, as the best which could have been instituted under the circumstances.

*Prof. White* remarked, that the attention of the profession having been recently directed to the investigation of uterine hæmatocele, he would briefly call the attention of the Society to a case now under his care.

*A Case of Retro Uterine Hœmatocele.*—On the 23d of June, called to see Mrs. W., of St. Louis, who was now staying at the house of a relative, living on Pearl street, in this City. The patient is 32 years old, married, and has had four mature pregnancies and several abortions. She has been flowing rather profusely during the last three weeks, and is now suffering severe pelvic pain with some abdominal tenderness. Pulse one hundred, with considerable febrile movement; dysenteric tenesmus was also present and added to her sufferings. Without instituting a vaginal examination, directed a teaspoon full of laudanum to be given by enema, mustard poultice to the abdomen, and enjoined quietude in the horizontal posture.

Next day, June 24th, found my patient much more comfortable, with the menorrhagia considerably diminished, and the dysenteric symptoms relieved. My suspicions that the urgent symptoms were due to the long journey performed whilst menstruating, and the fatigue consequent upon the care of her youngest child, now a little more than two years old, were confirmed. I, therefore, dismissed the case, merely prescribing quinine and iron as a tonic.

On the 3d of July, I was again sent for, and found Mrs. W. still flowing and suffering more severely from pain in the pelvis, with an increase of the abdominal tenderness and dysenteric efforts. Since my previous visits, she had had frequent recourse to the anodoyne enema which I had then recommended, and each time with temporary relief only. Upon making a digital examination, the os uteri was found crowded up anteriorly, and quite patulous. Carrying the finger backward, it encountered a large, elastic tumor, which occupied the whole of the posterior pelvic cavity. The sound was easily carried up three and a half or four inches into the uterus, with its concavity anteriorly directed. The abdominal parietus being thin, the point of the sound could be distinctly felt, by the finger of the left hand, in the supra pubic region. A rectal examination also discovered a large elastic tumor, filling the posterior pelvic space, and completely collapsing the rectum. Not having at hand an exploring trochar, I was unable to confirm, at that visit, my suspicions of the existence of retro-uterine hæmatocele. Although the patient had made frequent efforts at stool, the contents of the bowels had not been evacuated, and I directed the administration of an operative dose of castor oil, and promised to return and complete the diagnosis in a day or two.

Called, on the 5th, with my friend, Prof. Rochester. The oil had operated freely. The patient was now suffering severely from pain in the pelvic region, although she had had recourse to the laudanum injections for its relief. The rational symptoms, character of pain, and its location, were very like those produced by sudden retroflexion or retroversion of the uterus. There was more exhaustion, and, perhaps, also more abdominal tenderness than are usually present in cases of displacement. Since my last visit, the tumor had increased in size and density. It could be distinctly felt, on the right side, above the pelvis. The hand on the abdomen, with the fingers of the other hand in the vagina, passed it back and forth, and pressure with one hand was distinctly felt by the opposing hand. Its largest diameter was not less than seven inches, whilst it filled the posterior pelvic space from the floor of the pelvis upward.

Upon inserting a large exploring trochar into the lower segment of the tumor, and leaving the canula, for a few minutes, nothing escaped. After withdrawing the canula, however, it was found, upon blowing out its contents, to be filled with thick, dark blood, thus confirming my previous conjectures as to the nature of the contents of the tumor.

The pain was so severe, and the tumor so large, as to demand immediate relief, and exclude the hope of cure by the slow process of absorption, if, indeed, this course is ever to be adopted. The *manner* of evacuating the sac was then the only question to be decided in this instance. It ought to be said, in this connection, that the tumor had plainly increased in size during the last two days, and the pain in severity, and the patient begged for relief at any risk.

In a recent very able paper upon this subject, by J. Byrne, M. D., of Brooklyn, puncture through the rectum is recommended. Dr. B., who has written the best description of uterine hæmatacele to be found, so far as I know, in the English language, adduces many arguments in favor of rectal over vaginal openings. It is true, also, that, according to the statistics of M. Voisin, who has given us a resumé of French authors on this subject, the proportion of *spontaneous* ruptures were twice as great through the rectum as through the vagina. My own prejudice was, however, in favor of opening through the vagina. In the only cases upon which I have operated, one before and one subsequent to supperation, both of which recovered; free egress had been given to the contents of the sac through the vagina. Besides, if we puncture through the rectum, it necessarily involves the infliction of two wounds in the peritoneum, both of which are avoided by the vaginal outlet.

Again, it would, at least in this instance, have been found impossible to have removed the contents of the tumor through any puncture which I should feel warranted in making, through the rectum and the peritoneal cul de sac between it and the cavity of the hæmatocele.

I, therefore, in conformity with these views, undertook to give the patient immediate relief by passing a large trochar into the lower segment of the over distended sac through its vaginal covering. The canula used, measures a little more than three eights of an inch in its interior diameter. After withdrawing the trochar, notwithstanding the large size of the tube, only a little dark blood and a few small black coagula escaped. The uterine sound was then carried up through the canula and freely moved about in the cavity of the tumor, when two or three ounces of a similar fluid escaped. The tumor was however but slightly diminished in size or density, and it did not seem possible for its contents to be removed in this manner,

Believing it safer to make a free opening and completely evacuate and wash out the sac than to leave it in this condition, Simpson's uterotome was carried up through the puncture and opened in a lateral direction to fully one inch, and then withdrawn. It was then reintroduced, turned in the opposite direction and opened to fully one and one-half inches, thus upon its withdrawal, laying open the whole lower extremity of the tumor. A large amount of firm black coagula now escaped with immediate relief to all the urgent symptoms.

After allowing the patient to obtain a little much needed rest and take a little nourishment and brandy, a long flexible gum elastic tube was carried well up into the cavity of the tumor, and through it tepid water was injected until a large amount of its contents were washed out. The vaginal portion of the tumor now collapsed and its upper extremity could scarcely be felt in the right illiac region above the pubis. The patient was now quite comfortable, slept well the following night, and had some appetite.

July 25.—But little febrile movement succeeded the operation, the pulse at no time rising above one hundred, and the abdominal tenderness and pelvic pains have steadily diminshed.

The discharge from the sac has been, most of the time, quite free. At one time, the hemorrhage was so free, and the patient becoming so anæmic, I deemed it prudent to inject a strong solution of persulphate of iron. This was resorted to on two occasions, with the effect of greatly diminishing the flow. The daily use of warm water, or when the odor required, chloride of soda added, with cleanliness, constituted the local treatment. She has been taking freely of quinine and iron; her diet has been nutritious and stimulants have been freely administered since the operation, and is now quite convalescent. The opening in the posterior walls of the vagina has nearly closed, the discharge disappeared, and little evidence of the existence of the tumor can be felt with the finger in the vagina or rectum.

There is little time left for any remarks which the case may suggest.—It will be seen that the patient was at the age when this accident is most likely to occur, (32 years,) that she had been frequently pregnant and was at the time suffering from profuse menorrhagia.

In all the instances of hæmatocele, which have came under my observation, (this being the fourth,) there has been as in this instance, hypertrophy of the uterus, ascertained by internal measurement with the sound. There is probably congestion of the vessels of the uterus and not unlikely varicose condition of the vessels of the posterior uterine surface occompanying the increase in the size of the organ. It is not unlikely that the long journey from St. Louis to Buffalo, performedwhilst there was great temporary increase of this local congestion, and the long continued concussion of car riding, ruptured the over distended vessels and produced the effusion between the posterior walls of the organ and the peritoneal envelope.—The tumor, in this instance, attained an unusual size, and was formed gradually.

This case also shows the relief to be expected to the urgent symptoms by opening the sac, and the safety of making that opening through the vagina and dividing the tissues freely. It is not unlikely also, that the method of proceedure in making, the opening adopted in this instance from a combination of circumstances which accidently led to it, may prove a useful suggestion to future operators under similar circumstances.

*Prof. Rochester* observed, that this case reported by Prof. White, was the first he had seen; that though the disease was not new, yet the diagnosis of it was very new. Patients often speak of tumors in the groin which have disappeared; they are probably sometimes of this nature; small ones may be, and probably are, absorbed. Dr. Byrne advises to make the opening through the rectum; in this case, sufficiently large opening could not have been made without endangering the recto vaginal walls.

*Dr, Miner* spoke of the similarity in appearance and treatment, of hæmatocele and pelvic abscess. Knew of no means of positively distinguishing the one from the other, without exploration, as had already been remarked, and could see no objection to introducing common trocar into large accumulation of either blood or pus, when found in that vicinity.—Regarded the exploring trocar as too small to afford any very satisfactory evidence in some cases, since coagulated blood or pus, would not pass readily through it. Could not speak of hæmatocele from experience, but in pelvic abscess, preferred introducing trocar through the rectum, since the subsequent exit of accumulation was controlled by the sphincter, which was much more tolerable than constant discharge.

Thought there could be no danger in introducing even a very large trocar and canula, to which an exhausting pump might be applied and the contents drawn off and the cavity washed if necessary. Coagulated blood is sometimes not easily broken down, and he had no doubt as to the propriety and necessity of the operation as practiced by Prof. White, indeed had no opinion to expressd since he had no experience in the disease of which Dr. White had furnished an interesting report.

Dr. McKennon was proposed for membership.

Voted to adjourn to the first Tuesday evening in August.

J. F. MINER, Secretary.

